# Chromatic pupillometry isolation and evaluation of intrinsically photosensitive retinal ganglion cell-driven pupillary light response in patients with retinitis pigmentosa

**DOI:** 10.3389/fnhum.2023.1212398

**Published:** 2023-07-18

**Authors:** He Zhao, Hao Wang, Minfang Zhang, Chuanhuang Weng, Yong Liu, Zhengqin Yin

**Affiliations:** ^1^Southwest Hospital/Southwest Eye Hospital, Army Medical University, Chongqing, China; ^2^Key Lab of Visual Damage and Regeneration and Restoration of Chongqing, Chongqing, China

**Keywords:** advanced retinitis pigmentosa, chromatic pupillometry, intrinsically photosensitive retinal ganglion cells, postillumination pupil response, pupil kinetics

## Abstract

**Purpose:**

The pupil light response (PLR) is driven by rods, cones, and intrinsically photosensitive retinal ganglion cells (ipRGCs). We aimed to isolate ipRGC-driven pupil responses using chromatic pupillometry and to determine the effect of advanced retinitis pigmentosa (RP) on ipRGC function.

**Methods:**

A total of 100 eyes from 67 patients with advanced RP and 18 healthy controls (HCs) were included. Patients were divided into groups according to severity of visual impairment: no light perception (NLP, 9 eyes), light perception (LP, 19 eyes), faint form perception (FFP, 34 eyes), or form perception (FP, 38 eyes). Pupil responses to rod-weighted (487 nm, −1 log cd/m^2^, 1 s), cone-weighted (630 nm, 2 log cd/m^2^, 1 s), and ipRGC-weighted (487 nm, 2 log cd/m^2^, 1 s) stimuli were recorded. ipRGC function was evaluated by the postillumination pupil response (PIPR) and three metrics of pupil kinetics: maximal contraction velocity (MCV), contraction duration, and maximum dilation velocity (MDV).

**Results:**

We found a slow, sustained PLR response to the ipRGC-weighted stimulus in most patients with NLP (8/9), but these patients had no detectable rod- or cone-driven PLR. The ipRGC-driven PLR had an MCV of 0.269 ± 0.150%/s and contraction duration of 2.562 ± 0.902 s, both of which were significantly lower than those of the rod and cone responses. The PIPRs of the RP groups did not decrease compared with those of the HCs group and were even enhanced in the LP group. At advanced stages, ipRGC responses gradually became the main component of the PLR.

**Conclusion:**

Chromatic pupillometry successfully isolated an ipRGC-driven PLR in patients with advanced RP. This PLR remained stable and gradually became the main driver of pupil contraction in more advanced cases of RP. Here, we present baseline data on ipRGC function; we expect these findings to contribute to evaluating and screening candidates for novel therapies.

## Introduction

Retinitis pigmentosa (RP) has become most common irreversible blinding genetic eye disease threatening human visual health (GBD 2019 Blindness and Vision Impairment Collaborators, and Vision Loss Expert Group of the Global Burden of Disease Study, 2021). The treatment of RP has always been a focus of ophthalmic research. To date, many therapeutic strategies have demonstrated the potentiality to restore visual function in animal studies (Ahmed et al., 2021; Sharma and Jaganathan, 2021; Wagner et al., 2021). However, in clinical trials, both gene therapy and stem cell therapy have encountered challenges in their application to advanced RP patients (Gasparini et al., 2019; Nuzbrokh et al., 2021). Many research groups have focused on optogenetic strategies targeting the inner retina to restore visual function by directly activating the neural pathways from retinal ganglion cells to the visual cortex (Liu et al., 2016; Lu et al., 2020; Lindner et al., 2022). Therefore, objective and quantitative evaluation of the function of the inner retina, particularly the retinal ganglion cells, is crucial to research on therapies for advanced RP (Dias et al., 2018).

The pupil light response (PLR) is a convenient, objective, and non-invasive method of assessing subcortical visual pathway function (Belliveau et al., 2022). Chromatic pupillometry is a new PLR-based examination technique that can activate the pupil response induced by the corresponding pathway through light stimulation of a specific intensity and wavelength based on the photosensitive properties of different visual cells and then evaluate the function of the outer and inner retina (Schnapf et al., 1988; Park et al., 2010). For example, the rod-driven PLR can be induced by light stimulation below the threshold of cone cells and ipRGCs after dark adaptation because rhodopsin has a lower photosensitivity threshold than the opsins present in cones and ipRGCs (Okawa and Sampath, 2007). L-opsin, expressed in cones, is sensitive to long-wavelength light; thus, a cone-driven PLR can be elicited with light whose wavelength is too long to be absorbed by rhodopsin or melanopsin (Bowmaker and Dartnall, 1980). The pupil response driven by ipRGCs has time-domain characteristics different from those of rods and cones (Berson et al., 2002). When a bright short-wavelength light pulse begins, the initial transient pupillary response is a combination of responses activated by all photoreceptors (Güler et al., 2008; McDougal and Gamlin, 2010). As time passes and the extrinsic inputs from rod and cones decay rapidly, the intrinsic input from ipRGCs increases and mediates the postillumination pupil response (PIPR) (Adhikari et al., 2015; Park and McAnany, 2015), which consists of sustained pupil contraction after light offset. As a measure of ipRGC function, PIPR has been widely applied in glaucoma, optic neuropathy, diabetes and retinal dystrophy (Vugler et al., 2008a; Feigl et al., 2012; Kawasaki et al., 2014; Najjar et al., 2018). However, it is still a challenge to separate the ipRGC-driven PLR component from the mixed pupil response. Although activating only ipRGCs while screening out the outer retinal signal can be achieved *in vivo* in canines with loss-of-function mutations (Yeh et al., 2017) and pharmacologically blocked non-human primates (Gamlin et al., 2007), further study is still needed to establish a complete description of the isolated ipRGC-driven PLR. Therefore, this study was designed to provide a baseline reference for rational screening of treatment candidates for retinal prostheses or optogenetic therapy for end-stage RP and evaluation of visual function improvement before and after treatment.

Owing to a nearly complete loss of visual acuity, patients with advanced RP cannot be evaluated with standard visual field (VF) tests or standard full-field electroretinography (ffERG), and they are often assessed only by subjective psychophysical examination. Many studies suggest that chromatic pupillometry is more sensitive than standard visual electrophysiological examination for detecting retinal functional activity in the later stages of retinal degeneration (Park et al., 2010; Jacobson et al., 2011; Kardon et al., 2011; Lisowska et al., 2017; Rukmini et al., 2019; Krishnan et al., 2020; Yamamoto et al., 2023). In previous work, we demonstrated that chromatic pupillometry can quantitatively estimate retinal function in RP patients (He et al., 2018), and we used this method to evaluate the therapeutic effect of stem cell transplantation in clinical trials (Liu et al., 2017). Additionally, we found that high-intensity blue light can induce pupillary responses that are not easily attenuated (Zhang et al., 2021), even in patients with NLP. It has also been reported that long-duration white light stimuli induced a slow PLR in patients with severe Leber congenital amaurosis (LCA) (Charng et al., 2017), which was likely due to activation of the intrinsic circuit of ipRGCs. However, due to the limited sample size and study design, further research on ipRGC-driven PLR in patients with different stages of advanced RP is still needed.

In this study, we aimed to further isolate ipRGC-driven pupil responses by chromatic pupillometry in end-stage RP patients and to describe the characteristics of ipRGC-driven pupil responses in advanced RP patients, with the goal of providing background information and guidance for future treatment strategies targeting ganglion cells.

## Materials and methods

### Study population

The study comprised 100 eyes from 67 individuals diagnosed with advanced RP by three expert ophthalmologists from the Eye Institute of Southwest Hospital. All patients underwent routine ophthalmic examinations as follows: slit-lamp examination, best-corrected visual acuity (BCVA), chromatic fundus photography (CFP), fundus autofluorescence (FAF), optical coherence tomography (OCT), VF testing and ffERG examination. The clinical diagnosis of RP-related blindness was based on the following: characteristic bone-spicule pigmentation in the retina, extinguished scotopic and photopic responses on ffERG and concentric VF loss. The inclusion criteria for patients were as follows: the BCVA of the study eye was less than 3/60 or the visual field radius was less than 10 degrees around central fixation (legally blind) (Faiad et al., 2018). If both of the patient’s eyes met the inclusion criteria and the degree of visual impairment was consistent, both eyes were included; if there was a difference in visual impairment between the patient’s two eyes, the eye with worse vision was included.

**FIGURE 1:**
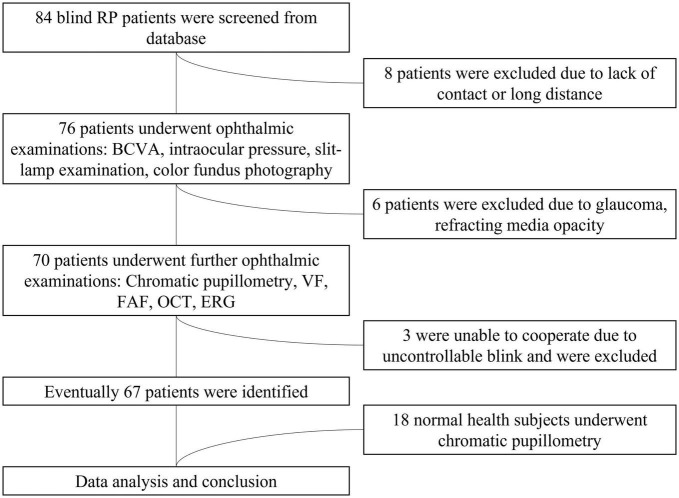
Flowchart of the patient selection process. RP, retinitis pigmentosa; BCVA, best-corrected visual acuity; VF, visual field; ERG, electroretinogram; FAF, fundus autofluorescence; OCT, optical coherence tomography.

We recruited 67 participants with advanced RP from a pool of 84 patients (see Figure 1). The study participants’ eyes were separated into four groups based on their BCVA levels. The four groups were delineated as no light perception (NLP), light perception (LP), hand motion or counting fingers (faint form perception, FFP), and 0.01–0.5 (form perception, FP). Eighteen additional healthy subjects (8 males, 10 females; average age ± SD: 32.94 ± 14.47 years) were enrolled as healthy controls for the chromatic pupillometry examination. Patients who had any other eye disease that could cause visual impairment, took any medication that affected the pupil or had dysfunctional pupils due to an iris disease were excluded from the study. This study was performed in accordance with the Declaration of Helsinki, and informed consent and permission were obtained. This research was authorized by the Ethics Committee of the First Affiliated Hospital of Army Medical University [(B) KY202221].

### Chromatic pupillometry

Pupillometry was performed with a commercially available visual surveillance system (Metrovision, France). The visual surveillance system included a stimulus-generating unit and a recording unit. The full-field stimuli were conducted by an LED-driven Ganzfeld bowl, which was set and controlled by a computer connected to the visual surveillance system to generate a given wavelength, intensity and duration. The pupil was scanned and measured by a high-resolution infrared camera with a 200 Hz refresh rate. Unavoidable blink-and-eye movement artifacts were initially removed by the automatic filter of the system’s built-in software, while other large unfilterable artifacts needed to be identified and removed manually in the later data processing phase.

### Light stimulation protocol

The original protocol for pupillary measurements was previously published (Zhang et al., 2021). However, to favor the melanopsin contribution to ipRGC activity, we referenced the protocols described in the current literature (Lorenz et al., 2012; Kawasaki et al., 2014; Adhikari et al., 2015) and modified the previously published stimulus protocol for this study. The duration of the stimulation was 1 s, and the recording time was 7.5 s. In each test, the unilateral eye was stimulated while the contralateral eye was patched. The light stimulation procedure included the following three tests: The first test (rod-weighted) used a dim blue light (487 ± 20 nm, −1 log cd/m^2^) stimulus after dark adaptation. The second test (cone-weighted) used a bright red light (630 ± 20 nm, 2 log cd/m^2^) stimulus following 2 min of photopic adaptation to room light. The third test (ipRGC-weighted) used a bright blue light (487 ± 20 nm, 2 log cd/m^2^) stimulus after dark adaptation. For each test, the measurements were repeated 3 times. For dark adaptation, the subject underwent at least 10 min of dark adaptation to ensure that the pupil diameter returned to the baseline level.

### Preprocessing and analysis of pupillometry

First, the baseline pupil diameter (D_0_) was determined as the mean initial pupil diameter over the first 5 s of recording in darkness. To reduce the influence of individual variation, the PLRs for each patient were measured as the relative change because pupillomotor responses are dependent on the baseline pupil diameter. Relative pupillary constriction (RPC) was calculated as follows:


$$\mathrm{RPC}\,\mathrm{at}\,\mathrm{time}\,\text{t}=\frac{{\text{D}}_{0}-\mathrm{pupil}\,\mathrm{diameter}\,\mathrm{at}\,\mathrm{time}\,\text{t}}{{\text{D}}_{0}}$$

**FIGURE 2:**
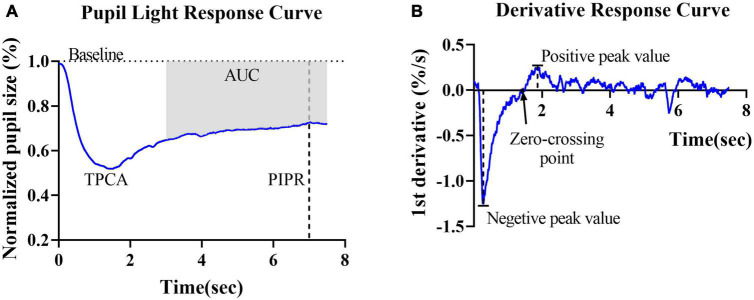
Graphic representation of the parameters obtained from the pupillary light reflex curve and derivative curve in healthy controls. **(A)** Mean PLR curve to blue light (487 ± 20 nm, 2 log cd/m^2^) in healthy controls. The baseline pupil diameter (*horizontal dashed line*), postillumination pupil response (PIPR) (*vertical dashed line*) and area under the curve (AUC) are indicated in the plot. **(B)** The average derivative of the pupil response curve shown in panel **(A)**. The negative peak value, positive peak value and first zero-crossing point (*black arrow*) are indicated in the plot.

Rod- and cone-weighted pupil responses were observed as transient PLRs, whereas the ipRGC-weighted response was observed as a sustained PLR, i.e., the PIPR. In this study, the PIPR was defined as the RPC at 6 s after light offset. The net PIPR was calculated as the difference between the amplitudes of the blue and red PIPRs. The area upon the curve (AUC) represented the average relative pupil size during 3–7.5 s postillumination (Figure 2A).

To further describe and investigate variations in contraction and dilation, the first derivative *d’(t)* of the relative pupil size was computed. For the derivative curves, negative values indicate the velocity of pupil contraction, and positive values indicate pupil dilation velocity. Two peak *d’(t)* values were identified: the maximal contraction velocity (negative peak value) and the maximal dilation velocity (positive peak value). The first zero-crossing point after the negative peak of a derivative curve indicated the end of the pupil contraction phase, i.e., the final timepoint used to compute contraction duration (Figure 2B).

### Statistical analysis

IBM SPSS Statistics version 22 (IBM Corporation, USA) was used to conduct the statistical analysis. First, normality of each variable was verified by the Shapiro−Wilk test. Sex differences across groups were tested by the chi-square test. The independent Samples t- test and Ordinary one-way analysis of variance (ANOVA) was conducted on normally distributed data. Otherwise, non-parametric methods were applied, including the Mann−Whitney U test and the Kruskal−Wallis test. Any *P*-value less than 0.05 was considered statistically significant. The *post hoc* analysis was Bonferroni corrected, and the adjusted *P*-value was set to *P_adj* < 0.05 in ANOVA and Kruskal−Wallis tests.

## Results

### Demography and routine ophthalmic examination of retinitis pigmentosa patients

This study included a total of 100 eyes from 67 patients with advanced RP. Based on their BCVA values, the patients’ eyes were separated into four groups. The demographic details of the participants are shown in Table 1. The groups exhibited no significant differences in age (*F* = 1.350, *P* = 0.2664, ANOVA) or sex (chi-square value = 3.032, *P* = 0.3867, chi-square test).

**TABLE 1:** Demographics of patients with advanced RP.

	NLP group	LP group	FFP group	FP group
Eyes/patients	9/6	19/12	34/23	38/26
Sex (male/female)	3/3	8/4	10/13	17/9
Age (mean ± SD)	46.33 ± 11.43	42.58 ± 12.15	45.23 ± 11.39	40.04 ± 7.33
BCVA	NLP	LP	HM or CF	0.01–0.5
Visual field radius	0°	0°	0°	<10°

SD, standard deviation; NLP, no light perception, LP, light perception, HM, hand movement, CF, counting finger, BCVA, best-corrected visual acuity.

**FIGURE 3:**
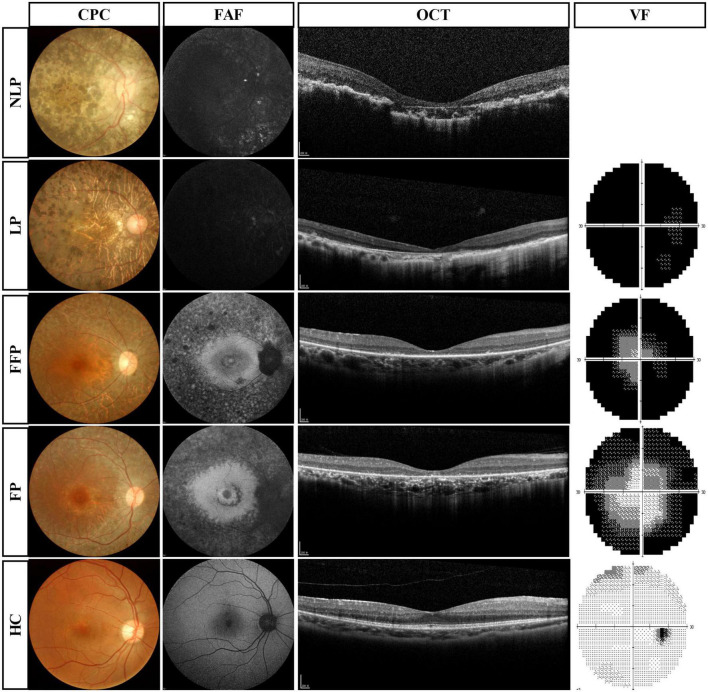
Routine ophthalmologic examinations of typical patients with advanced RP and healthy subjects. Subjects are ranked vertically in ascending order of severity of visual impairment, and different types of examination are shown as columns. VF results were absent in patients with NLP. RP, retinitis pigmentosa; NLP, no light perception; LP, light perception; FFP, faint form perception; FP, form perception; HC, healthy controls; CFP, color fundus photography; FAF, fundus autofluorescence; OCT, optical coherence tomography; VF, visual field.

The results of routine ophthalmologic examinations in patients with advanced RP and in healthy control subjects are shown in Figure 3. The CFP results showed retinal atrophy and bone spicule pigmentation in the retinas of patients, and the FAF showed that RP patients progressively lost retinal pigment epithelium. Macular OCT showed reduced retinal thickness and disordered structural hierarchy, and VF examination indicated that the visual field of patients with advanced RP was reduced or even completely lost.

### The IpRGC-driven pupillary light response was isolated in patients with advanced RP and no light perception

**FIGURE 4:**
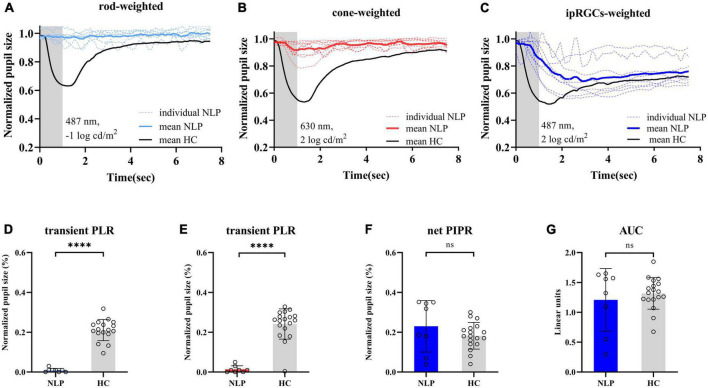
Pupil light response curve recorded by three light stimulus protocols and statistical analysis of contraction amplitude in RP patients with no light perception and healthy controls. **(A–C)** Following the rod-weighted stimulus (487 ± 20 nm, –1 log cd/m^2^), cone-weighted stimulus (630 ± 20 nm, 2 log cd/m^2^), and ipRGC-weighted stimulus (487 ± 20 nm, 2 log cd/m^2^), normalized PLR curves of RP individuals (dashed sky blue, red and royal blue, respectively) and the mean RP PLR (solid sky blue, red and royal blue, respectively) were plotted. The gray areas represent the duration of the light stimulus, and the solid black lines represent healthy controls’ average PLR curves. **(D,E)** Comparison of transient PLR between RP patients (blue bars for rod-weighted, red bars for cone-weighted) and healthy controls (gray bars with standard deviations) following rod- and cone-weighted stimulus. **(F,G)** Comparison of net PIPR and AUC for healthy controls (gray bars with standard deviations) and RP patients (blue bars with standard deviations) following ipRGC -weighted stimulus. The sample sizes were 8 (NLP) and 18 (HC) eyes for three light stimuli. PLR, ipRGCs, intrinsically photosensitive retinal ganglion cells; RP, retinitis pigmentosa; NLP, no light perception; HC, healthy controls; PIPR, postillumination pupil response; AUC, area under the curve. ****P < 0.0001. ns: not significant.

To observe the pure ipRGC-driven PLR without extrinsic signals originating in the outer retinal rod and cone photoreceptors, the pupillomotor function of 9 eyes of 6 patients in the NLP group was examined. These eyes of patients with advanced RP all had NLP vision and extinguished ERG readings. Average pupil response curves from NLP patients are shown in Figure 4. In all cases, there was no discernible PLR in response to the rod-weighted stimulus, in contrast to the large-amplitude transient PLR obtained in normal eyes (Figure 4A). Under the cone-weighted stimulus condition, 6 eyes had no recordable pupil contraction in response to red light stimulation. Minor pupillary contractions were observed in 3 eyes (Figure 4B), but the patients reported no light perception during the examination. In response to the bright blue ipRGC-weighted stimulus, 8 eyes showed slow and sustained pupillary contraction (Figure 4C) one eye did not due to an unrecognizable pupil tremor. The net PIPR amplitude of NLP patients was 0.230 ± 0.128, whereas the net PIPR of healthy subjects was 0.156 ± 0.084. Likewise, the AUC of NLP patients was greater than that of the control group, but there was no significant difference in net PIPR amplitude or AUC between NLP patients and healthy controls (*U* = 51, *P* = 0.2321; *U* = 68, 0.7438, respectively; Mann−Whitney test) (Figures 4F, G). Unsurprisingly, due to the lack of input from photoreceptors, the transient PLR amplitude was significantly lower in NLP patients than in healthy controls with rod- and cone-weighted stimuli (both U = 0, *P* < 0.0001; Mann−Whitney test) (Figures 2D, E). Our data showed that the shape of the PLR solely driven by ipRGCs did not match the normal human PLR evoked by any stimulus pattern but was similar to the shape of the PLR in primates after pharmacological blockade of photoreceptor inputs (Schnapf et al., 1988). These data suggested that, in the absence of extrinsic signal input from the outer retina, melanopsin-expressing ipRGCs produced a slow and sustained response to bright blue stimuli and that this response was isolated in NLP patients with advanced RP.

### The IpRGC-driven pupillary light response had unique pupillary kinetics different from the cone and rod responses

**FIGURE 5:**
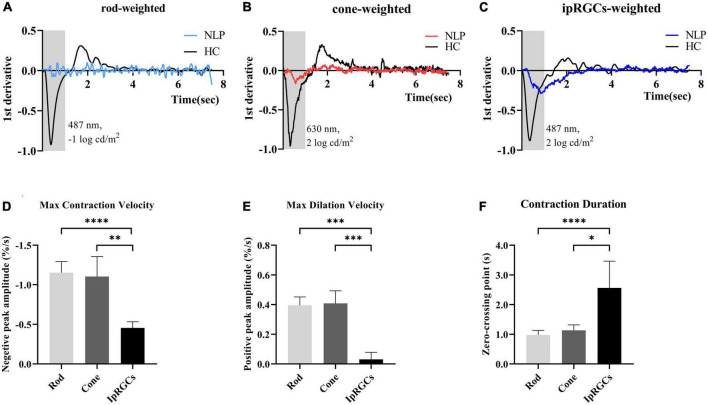
Analysis of the derivative of the pupil response curves in NLP patients with advanced RP and healthy controls. **(A–C)** The mean derivative responses plotted for the pupil recordings of rod, cone and ipRGC responses in RP patients (solid sky blue, red and royal blue, respectively) and healthy controls (solid black). **(D)** Comparison of the maximum contraction velocity of the ipRGC response in RP patients (*N* = 8) and that of the rod or cone response in healthy controls (*N* = 18). **(E)** Statistical analysis of the maximum dilation velocity of the ipRGC response in RP patients (*N* = 8) and that of the rod or cone response in healthy controls (*N* = 18). **(F)** Statistical analysis of the contraction duration of the ipRGC response in RP patients (*N* = 8) and that of the rod or cone response in normal subjects (*N* = 18). RP, retinitis pigmentosa; ipRGCs, intrinsically photosensitive retinal ganglion cells. ****P < 0.0001, ***P < 0.001, **P < 0.01, and *P < 0.05.

To further detail the ipRGC-driven pupillary light reflex, we investigated the pupillary contraction and dilation kinetics by analyzing the derivative of the PLR (Figures 5A–C). Unlike responses driven by cone and rod cells, we found that ipRGC-driven pupil contraction was very slow after light stimulus onset and that the pupil continued to contract after light offset, not stopping immediately. The analysis of contraction kinetics suggested that the maximal contraction velocity (MCV) of the ipRGC response was considerably less than those of the rod and cone responses (rods vs. ipRGCs, Test statistic = −23.28, *P_adj* < 0.0001; cones vs. ipRGCs, Test statistic = −19.72, *P_adj* = 0.0013; Kruskal−Wallis test) (Figure 5D), and the contraction duration was prolonged (rods vs. ipRGCs, Test statistic = −24.60, *P_adj*<0.0001; cone vs. ipRGCs, Test statistic = −15.33, *P_adj* = 0.0184; Kruskal−Wallis test) (Figure 5E). The rapid dilation phase of the ipRGC response was not recorded in patients with NLP until the end point of the observation, usually occurring in first 3 s after light offset in healthy controls. Instead, a persistent contraction of the pupil was recorded, which resulted in the MDV of the ipRGC response being much smaller than those of rod and cone cells (rods vs. ipRGCs, Test statistic = 21.47, *P_adj* = 0.0004; cones vs. ipRGCs, Test statistic = 21.53, *P_adj* = 0.0004; Kruskal−Wallis test) (Figure 5F). To investigate whether this type of response varied with disease progression, we further compared ipRGC-driven PLRs among the four groups of patients with advanced RP.

### IpRGC contribution to pupil contraction gradually increases with more advanced RP

**FIGURE 6:**
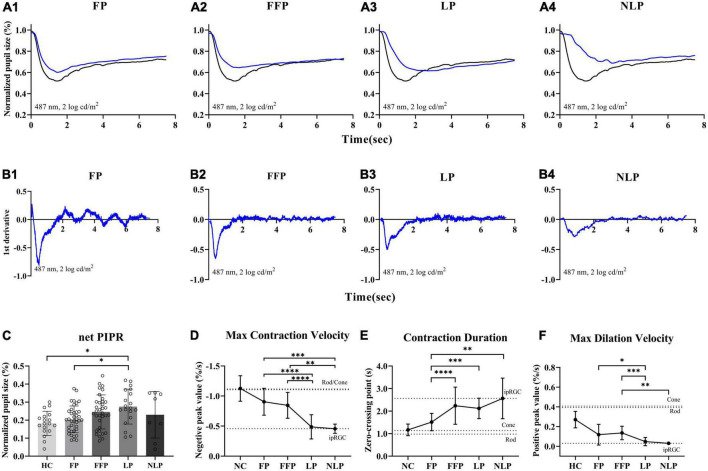
Comparison of ipRGC-driven pupil response in four groups of patients with advanced RP. **(A1–4)** Following testing with an ipRGC-weighted stimulus (487 ± 20 nm, 2 log cd/m^2^), average normalized PLR curves of RP patients (blue) and healthy controls (black) were plotted. **(B1–4)** Average derivative responses plotted for the pupil recordings of four groups of RP patients. **(C)** Statistical analysis of net PIPR among RP patients. Standard deviations are shown with error bars. **(D–F)** Plot showing the difference and variation tendency of contraction and dilation kinetics in the four RP groups. RP, retinitis pigmentosa; ipRGCs, intrinsically photosensitive retinal ganglion cells; PIPR, postillumination pupil response; MCV, maximal contraction velocity; MDV, maximal dilation velocity. ****P < 0.0001, ***P < 0.001, **P < 0.01, and *P < 0.05.

Considering that ipRGC-driven pupillary responses were observed in a small proportion of patients with the most severe RP, we wanted to determine whether this pupil response is common in more advanced RP patients and asked whether the function of ipRGCs is affected in the pathological process of RP. Therefore, we recorded and analyzed the pupil response to ipRGC-weighted stimulation in advanced RP patients with varying degrees of visual impairment. Apparent pupil responses were detected in all studied eyes in the NLP, LP, FFP, and FP groups. The average pupil response curves of the four groups are shown in Figure 6. The PLR metrics are detailed in Table 2. The sustained PLR was recorded in all groups under the ipRGC-weighted stimulus condition (Figure 6A). The net PIPRs of the four RP groups, NLP, LP, FFP, and FP, were 0.230 ± 0.129, 0.275 ± 0.097, 0.245 ± 0.094, and 0.204 ± 0.074, respectively. There were significant differences among the net PIPRs of the NLP, LP, FFP, FP and HC groups (*F* = 3.615, *P* = 0.0082; one-way ANOVA). A According to the results of a *post hoc* analysis with a Bonferroni correction, the net PIPR was significantly greater in the LP than in the FP group or the control group (LP vs. FP, Test statistic = 0.0709, *P*_adj = 0.0420; LP vs. HC, Test statistic = 0.0928, *P*_adj = 0.0168; one-way ANOVA), and the net PIPRs observed in the FFP and FP groups were not significantly different from those observed in the control group (FFP vs. HC, Test statistic = 0.0632, *P*_adj = 0.1151; FP vs. HC, Test statistic = 0.0218, *P*_adj = 0.7694, respectively; one-way ANOVA) (Figure 6C). The above results indicate that none of the RP groups (NLP, LP, FFP, or FP) had a net PIPR smaller than the normal amplitude of 0.182 ± 0.067. The derivative curve that further describes the variation in relative pupil size is shown in Figure 6B. Through the analysis of the derivative response parameters, we found that with the progression of visual impairment, the pupil contract velocity gradually decreased, and the MCVs of the groups without form perception were significantly less than those of the groups with form perception. The MCVs of the NLP and LP groups were similar to each other and lower than those of the FFP and FP groups, respectively (NLP vs. FFP, Test statistic = 42.32, *P_adj* = 0.0020; NLP vs. FP, Test statistic = 49.13, *P_adj* = 0.0002; LP vs. FFP, Test statistic = 36.87, *P_adj* < 0.0001; LP vs. FP, Test statistic = 43.68, *P_adj* < 0.0001; Kruskal−Wallis test) (Figure 6D). The contraction durations in the NLP, LP and FFP groups were similar to each other and longer than those of the FP group (NLP vs. FP, Test statistic = 44.01, *P_adj* = 0.0013; LP vs. FP, Test statistic = 33.56, *P_adj* = 0.0002; FFP vs. FP, Test statistic = 34.04, *P_adj* < 0.0001; Kruskal−Wallis test) (Figure 6E). The rapid dilation phase in the NLP and LP groups was absent, and the MDVs of these two groups were lower than those recorded for the FFP group (NLP vs. FFP, Test statistic = −42.90, *P_adj* = 0.0019; LP vs. FFP, Test statistic = −34.37, *P_adj* = 0.0002; Kruskal−Wallis test) and tended to be lower than those in the FP group (NLP vs. FP, Test statistic = −30.61, *P_adj* = 0.0566; LP vs. FP, Test statistic = −22.07, *P_adj* = 0.0361; Kruskal−Wallis test) (Figure 6F). The above results indicated that with outer retinal degeneration, the rapid transient pupillary response gradually vanished and was replaced by a slow sustained pupillary response to light, i.e., an ipRGC-driven response.

**TABLE 2:** Summary of PLR metrics.

PLR metrics	Max RPC	Net PIPR	AUC	MVC	Contraction duration	MDV
HC group	0.497 ± 0.035	0.156 ± 0.084	1.175 ± 0.578	−1.132 ± 0.222	1.156 ± 0.258	0.265 ± 0.080
NLP group	0.269 ± 0.150	0.230 ± 0.128	1.509 ± 0.427	−0.456 ± 0.076	2.562 ± 0.902	0.030 ± 0.047
LP group	0.338 ± 0.131	0.275 ± 0.097	1.298 ± 0.428	−0.488 ± 0.202	2.119 ± 0.454	0.066 ± 0.049
FFP group	0.397 ± 0.086	0.247 ± 0.094	1.260 ± 0.320	−0.874 ± 0.291	2.238 ± 0.824	0.136 ± 0.068
FP group	0.416 ± 0.088	0.211 ± 0.081	1.384 ± 0.128	−0.905 ± 0.224	1.512 ± 0.386	0.112 ± 0.111
Test statistic	37.97	3.615	2.27	57.73	57.38	44.24
d/f	4	4	4	4	4	3
*P*-value	<0.0001	0.0082	0.0311	<0.0001	<0.0001	<0.0001

PLR, pupil light response, RPC, relative pupillary constriction, AUC, area under curve, MCV, maximal contraction velocity, MDV, maximal dilation velocity, PIPR, postillumination pupil response, HC, healthy control, d/f, degrees of freedom.

**FIGURE 7:**
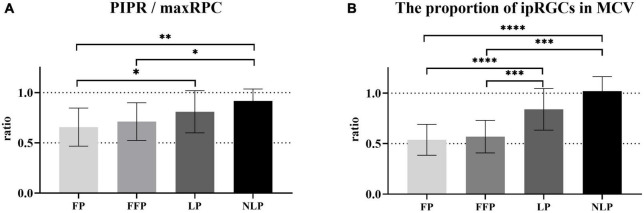
Comparison of the contributions of ipRGCs to the PLR in four groups of patients with advanced RP. **(A)** Bar chart plotting the average value of PIPR/maxRPC in the NLP, LP, FFP, and FP groups. **(B)** The ratio of the ipRGC response MCV to the MCVs of the NLP, LP, FFP, and FP groups. Interquartile ranges are shown with error bars. RP, retinitis pigmentosa; ipRGCs, intrinsically photosensitive retinal ganglion cells; PIPR, postillumination pupil response; RPC, relative pupillary constriction; MCV, maximal contraction velocity. ****P < 0.0001, ***P < 0.001, **P < 0.01, and *P < 0.05.

To determine how ipRGCs contribute to the PLR in RP patients at different stages, we first compared the ratio of sustained contraction amplitude to total contraction amplitude among groups. As shown in Figure 7A, we found that as the patient’s visual impairment worsened, the ratio of PIPR to the maxRPC gradually increased. Overall, the values of PIPR/maxRPC in the NLP, LP, and FFP groups were considerably greater than those in the HC group (H = 55.51; *P_adj* < 0.0001, *P_adj* = 0.0002, *P_adj* = 0.0177, respectively; Kruskal−Wallis test). When compared among the four RP groups, the value of PIPR/maxRPC was found to be larger in the NLP group than in the FFP and FP groups (NLP vs. FFP, Test statistic = 33.56, *P_adj* = 0.0394; NLP vs. FFP, Test statistic = 343.25, *P_adj* = 0.0021; Kruskal−Wallis test), but there was no significant difference from that of the LP group (Test statistic = 16.72, *P_adj* > 0.9999; Kruskal−Wallis test). In addition, the PIPR/maxRPC ratios of the LP groups were significantly higher than those of the FP group (Test statistic = 26.53, *P_adj* = 0.0124; Kruskal−Wallis test).

Next, considering that the PLR in the initial stage is a mixed reaction of ipRGCs and outer retinal photoreceptors, we also compared the baseline contraction velocity of the isolated ipRGC response with the MCV of each group. Generally, the proportion of ipRGC responses gradually increased in advanced RP patients. As shown in Figure 7B, there was no statistically significant difference between the ratio of the NLP group and that of the LP group, both of which were significantly greater than those in the other groups (NLP vs. FFP, Test statistic = 47.57, *P_adj* = 0.0004; NLP vs. FP, Test statistic = 53.43, *P_adj* < 0.0001; LP vs. FFP, Test statistic = 34.72, *P_adj* = 0.0002; LP vs. FP, Test statistic = 40.58, *P_adj* < 0.0001; Kruskal−Wallis test). The ratio of the FFP group did not differ substantially from the FP group (Test statistic = 5.86, *P_adj* > 0.9999; Kruskal−Wallis test). The above results indicate that ipRGC-driven PLR gradually became the main component of PLR in patients with advanced RP. The increasing contribution of ipRGCs seemed to be particularly pronounced in RP patients who had lost their form perception.

## Discussion

In this study, we demonstrated that it is possible to isolate ipRGCs’ response in advanced RP with a simple chromatic pupillometry procedure. Our data demonstrated that this pupil response was characterized by slow kinetics and was preserved or even enhanced in more severe outer retinal degeneration. Additionally, we showed that, as the disease progressed, ipRGCs gradually became the main driver of pupil contraction in patients with advanced RP.

Rods, cones, and melanopsin-expressing ipRGCs all contribute to the pupil light reflex. Due to different firing patterns, cones and rods mainly drive transient PLRs, while ipRGCs mainly drive a sustained PLR (Dacey et al., 2005; Gamlin et al., 2007). In this study, we used a bright blue light (487 ± 20 nm, 2 log cd/m^2^) stimulus to demonstrate and distinguish the activity of ipRGCs. Although the intensity of light stimulation can also induce PLR driven by residual rods, once the light is turned off, the input signal from the rods will decay rapidly. Therefore, consistent with previous peer-reviewed research (Park et al., 2011; Adhikari et al., 2015), the 6 s PIPR and the AUC at 3–7.5 s postillumination as used in this study can be used to evaluate the intrinsic signal of melanopsin-expressing ipRGCs. In this study, patients with NLP lacked photoreceptor inputs to the afferent pathway for the PLR due to severe degeneration of the outer retina. We believe that the slow and sustained pupil response described in Figure 4C was primarily driven by the intrinsic signaling of ipRGCs. A chromatic pupillometry study in patients with LCA (Charng et al., 2017) found a slow PLR induced by long-duration stimuli that had a distinct waveform that did not match normal PLR, a finding that is consistent with our study. However, due to the small sample size, that study did not report differences in patients with various severities of visual acuity.

As a marker of ipRGC activity, how does this unique pupillary response change during RP progression? In this study, we found that the ipRGC response was not attenuated in patients with varying degrees of visual impairment (Figure 3A). We were also surprised to find that ipRGC responses were enhanced in eyes with only light perception. Similarly, in a cohort of patients with RP caused by CRB1 mutations, the patients’ PIPR was larger than that in the control groups (Stingl et al., 2019). This could be explained by several phenomena. First, the structure and function of the inner retina, including ipRGCs, are preserved relative to the outer retina in RP, as evidenced by morphological studies of severe RP in humans showing the presence of a large proportion of bipolar and ganglion cells (Santos et al., 1997; Humayun et al., 1999; Yoon and Yu, 2018). Meanwhile, ipRGCs tend to survive due to their resistance to injury (Vugler et al., 2008b; Cui et al., 2015). Previous studies in animal models of retinal degeneration have demonstrated that the number of melanopsin-expressing ipRGCs does not decrease significantly as the disease progresses (Li et al., 2012; Esquiva et al., 2013). Another explanation might be the loss of the inhibitory effect of s-cones on ipRGCs (Spitschan et al., 2014). Moreover, another possible reason is the extensive rewiring of the retinal network in the late stages of retinal remodeling that occurs with the loss of normal photoreceptor input (Jones et al., 2016; Pfeiffer et al., 2020). Considering that ipRGCs possess a characteristic wide dendritic arbor, widespread neurite sprouting across all surviving retinal neurons and the creation of new synaptic contacts cause retinal function and circuit reorganization that may be the cause of signal amplification (Atkinson et al., 2013; Semo et al., 2016).

With the advent and rapid development of optogenetics, new therapies for visual regeneration are quickly evolving from proofs of concept to clinically adopted practices. Four research groups have already registered clinical trials of optogenetic therapy for patients with retinal degeneration (NCT03326336, NCT04945772, NCT02556736, and NCT04278131). Chromatic pupillometry allows for non-invasive and objective measurement of sustained retinal function. This technology can be used to track the course of natural diseases or the effectiveness of treatment interventions for retinal degenerative diseases. There have been several clinical trials using the PLR as an indicator to evaluate visual function rescue (Bennett et al., 2016; Lisowska et al., 2017; Liu et al., 2017). Furthermore, as an innovative and simple diagnostic technique, chromatic pupillometry may also be beneficial in the screening of patients as candidates for novel therapies. For example, the response to rod and cone stimulation suggests the retention of functional retinal circuits, and cell transplantation therapy and optogenetic intervention targeting bipolar cells may help improve visual function. The pupillary response of rods or cones has disappeared for blind people with end-stage RP; therefore, it is crucial to determine whether RGCs still function, which represent the retina’s final connection to the brain, are still functioning. The third-level retinal neurons represented by ipRGCs appear to respond to ipRGC-weighted stimulation, indicating that they are still functionally alive and still have the capacity to support thea retinal prosthesis or undergo optogenetic therapy.

The limitations of this study are the small sample size of recruited patients and the non-normal distribution of the data. The inclusion of two eyes from the same patient has an impact on the independence of observations. A better choice may be to include only one eye per patient, which can be implemented in subsequent large-scale multicenter clinical studies. It is generally believed that the rod-driven PLR is usually evoked by blue light stimuli in the low-intensity range (- 3 and - 2 log cd/m^2^) after dark adaptation, but our scheme fails to accurately evaluate rod function. In addition, more objective eye tests could have been included to investigate their relationship to the PLR. Pupil photography also has limitations in patients with low vision and blindness due to extreme nystagmus and fixation difficulties that make recording challenging, which ultimately led to complete data collection failure in one participant. More effective recording techniques are worth further development.

## Conclusion

We successfully recorded ipRGC-driven PLR in patients with severe visual impairments by chromatic pupillometry and showed that this method can detect the function of ipRGCs in patients with advanced RP. Chromatic pupillometry is a promising technique for evaluating retinal function in individuals with severe visual impairments and can be used as a precious tool in the research and development of novel therapies for restoring sight to people with visual impairments.

## Data availability statement

The raw data supporting the conclusions of this article will be made available by the authors, without undue reservation.

## Ethics statement

The studies involving human participants were reviewed and approved by the Ethics Committee of the First Affiliated Hospital of Army Medical University. The patients/participants provided their written informed consent to participate in this study.

## Author contributions

HZ: research design and manuscript writing. HZ and HW: data collection and analysis. YL and ZY: research guiding. MZ and CW: subjects collection. All authors contributed to the article and approved the submitted version.

## References

[B1] Adhikari P. Zele A. J. Feigl B. (2015). The Post-Illumination Pupil Response (PIPR). *Invest. Ophthalmol. Vis. Sci.* 56 3838–3849. 10.1167/iovs.14-16233 26066752

[B2] Ahmed I. Johnston R. Jr. Singh M. S. (2021). Pluripotent stem cell therapy for retinal diseases. *Ann. Trans. Med.* 9:1279. 10.21037/atm-20-4747 PMC8421932 34532416

[B3] Atkinson C. L. Feng J. Zhang D. Q. (2013). Functional integrity and modification of retinal dopaminergic neurons in the rd1 mutant mouse: Roles of melanopsin and GABA. *J. Neurophysiol.* 109 1589–1599. 10.1152/jn.00786.2012 23255724

[B4] Belliveau A. P. Somani A. N. Dossani R. H. (2022). *Pupillary light reflex.* Treasure Island, FL: StatPearls. 30725865

[B5] Bennett J. Wellman J. Marshall K. A. McCague S. Ashtari M. DiStefano-Pappas J. (2016). Safety and durability of effect of contralateral-eye administration of AAV2 gene therapy in patients with childhood-onset blindness caused by RPE65 mutations: A follow-on phase 1 trial. *Lancet* 388 661–672. 10.1016/S0140-6736(16)30371-3 27375040 PMC5351775

[B6] Berson D. M. Dunn F. A. Takao M. (2002). Phototransduction by retinal ganglion cells that set the circadian clock. *Science* 295 1070–1073. 10.1126/science.1067262 11834835

[B7] Bowmaker J. K. Dartnall H. J. (1980). Visual pigments of rods and cones in a human retina. *J. Physiol.* 298 501–511. 10.1113/jphysiol.1980.sp013097 7359434 PMC1279132

[B8] Charng J. Jacobson S. G. Heon E. Roman A. J. McGuigan D. B. Sheplock R. (2017). Pupillary light reflexes in severe photoreceptor blindness isolate the melanopic component of intrinsically photosensitive retinal ganglion cells. *Invest. Ophthalmol. Vis. Sci.* 58 3215–3224. 10.1167/iovs.17-21909 28660274 PMC5490362

[B9] Cui Q. Ren C. Sollars P. J. Pickard G. E. So K. F. (2015). The injury resistant ability of melanopsin-expressing intrinsically photosensitive retinal ganglion cells. *Neuroscience* 284 845–853. 10.1016/j.neuroscience.2014.11.002 25446359 PMC4637166

[B10] Dacey D. M. Liao H. W. Peterson B. B. Robinson F. R. Smith V. C. Pokorny J. (2005). Melanopsin-expressing ganglion cells in primate retina signal colour and irradiance and project to the LGN. *Nature* 433 749–754. 10.1038/nature03387 15716953

[B11] Dias M. F. Joo K. Kemp J. A. Fialho S. L. da Silva Cunha A. Jr. Woo S. J. (2018). Molecular genetics and emerging therapies for retinitis pigmentosa: Basic research and clinical perspectives. *Prog. Retin. Eye Res.* 63 107–131. 10.1016/j.preteyeres.2017.10.004 29097191

[B12] Esquiva G. Lax P. Cuenca N. (2013). Impairment of intrinsically photosensitive retinal ganglion cells associated with late stages of retinal degeneration. *Invest. Ophthalmol. Vis. Sci.* 54 4605–4618. 10.1167/iovs.13-12120 23766478

[B13] Faiad Y. Khoury B. Daouk S. Maj M. Keeley J. Gureje O. (2018). Frequency of use of the International Classification of Diseases ICD-10 diagnostic categories for mental and behavioural disorders across world regions. *Epidemiol. Psychiatr. Sci.* 27 568–576. 10.1017/S2045796017000683 29117869 PMC6999009

[B14] Feigl B. Zele A. J. Fader S. M. Howes A. N. Hughes C. E. Jones K. A. (2012). The post-illumination pupil response of melanopsin-expressing intrinsically photosensitive retinal ganglion cells in diabetes. *Acta Ophthalmol.* 90 e230–e234. 10.1111/j.1755-3768.2011.02226.x 21883986

[B15] Gamlin P. D. McDougal D. H. Pokorny J. Smith V. C. Yau K. W. Dacey D. M. (2007). Human and macaque pupil responses driven by melanopsin-containing retinal ganglion cells. *Vis. Res.* 47 946–954. 10.1016/j.visres.2006.12.015 17320141 PMC1945238

[B16] Gasparini S. J. Llonch S. Borsch O. Ader M. (2019). Transplantation of photoreceptors into the degenerative retina: Current state and future perspectives. *Prog. Retin. Eye Res.* 69 1–37. 10.1016/j.preteyeres.2018.11.001 30445193

[B17] GBD 2019 Blindness and Vision Impairment Collaborators, and Vision Loss Expert Group of the Global Burden of Disease Study (2021). Causes of blindness and vision impairment in 2020 and trends over 30 years, and prevalence of avoidable blindness in relation to VISION 2020: The right to sight: An analysis for the Global Burden of Disease Study. *Lancet Glob. Health* 9 e144–e160. 10.1016/S2214-109X(20)30489-7 33275949 PMC7820391

[B18] Güler A. D. Ecker J. L. Lall G. S. Haq S. Altimus C. M. Liao H.-W. (2008). Melanopsin cells are the principal conduits for rod-cone input to non-image-forming vision. *Nature* 453 102–105. 10.1038/nature06829 18432195 PMC2871301

[B19] He Y. Tang H. Wang G. Ren B. Wang Y. Liu Y. (2018). Correlation between transient pupillary light reflex and retinal function impairment in patients with retinitis pigmentosa. *J. Ophthalmol.* 2018:2519375. 10.1155/2018/2519375 30622818 PMC6304905

[B20] Humayun M. S. Prince M. de Juan E. Barron Y. Moskowitz M. Klock I. B. (1999). Morphometric analysis of the extramacular retina from postmortem eyes with retinitis pigmentosa. *Invest. Ophthalmol. Vis. Sci.* 40 143–148. 9888437

[B21] Jacobson S. G. Cideciyan A. V. Aleman T. S. Sumaroka A. Roman A. J. Swider M. (2011). Human retinal disease from AIPL1 gene mutations: Foveal cone loss with minimal macular photoreceptors and rod function remaining. *Invest. Ophthalmol. Vis. Sci.* 52 70–79. 10.1167/iovs.10-6127 20702822

[B22] Jones B. W. Pfeiffer R. L. Ferrell W. D. Watt C. B. Marmor M. Marc R. E. (2016). Retinal remodeling in human retinitis pigmentosa. *Exp. Eye Res.* 150 149–165. 10.1016/j.exer.2016.03.018 27020758 PMC5031517

[B23] Kardon R. Anderson S. C. Damarjian T. G. Grace E. M. Stone E. Kawasaki A. (2011). Chromatic pupillometry in patients with retinitis pigmentosa. *Ophthalmology* 118 376–381. 10.1016/j.ophtha.2010.06.033 20869119

[B24] Kawasaki A. Collomb S. Leon L. Munch M. (2014). Pupil responses derived from outer and inner retinal photoreception are normal in patients with hereditary optic neuropathy. *Exp. Eye Res.* 120 161–166. 10.1016/j.exer.2013.11.005 24275502

[B25] Krishnan A. K. Jacobson S. G. Roman A. J. Iyer B. S. Garofalo A. V. Heon E. (2020). Transient pupillary light reflex in CEP290- or NPHP5-associated Leber congenital amaurosis: Latency as a potential outcome measure of cone function. *Vis. Res.* 168 53–63. 10.1016/j.visres.2020.01.006 32088401 PMC7068155

[B26] Li Y. Li C. Chen Z. He J. Tao Z. Yin Z. Q. (2012). A microRNA, mir133b, suppresses melanopsin expression mediated by failure dopaminergic amacrine cells in RCS rats. *Cell. Signall.* 24 685–698. 10.1016/j.cellsig.2011.10.017 22101014

[B27] Lindner M. Gilhooley M. J. Hughes S. Hankins M. W. (2022). Optogenetics for visual restoration: From proof of principle to translational challenges. *Prog. Retin. Eye Res.* 91:101089. 10.1016/j.preteyeres.2022.101089 35691861

[B28] Lisowska J. Lisowski L. Kelbsch C. Maeda F. Richter P. Kohl S. (2017). Development of a chromatic pupillography protocol for the first gene therapy trial in patients with CNGA3-linked achromatopsia. *Invest. Ophthalmol. Vis. Sci.* 58 1274–1282. 10.1167/iovs.16-20505 28241315

[B29] Liu M. Dai J. Liu W. Zhao C. Yin Z. Q. (2016). Overexpression of melanopsin in the retina restores visual function in Royal College of Surgeons rats. *Mol. Med. Rep.* 13 321–326. 10.3892/mmr.2015.4549 26572076

[B30] Liu Y. Chen S. J. Li S. Y. Qu L. H. Meng X. H. Wang Y. (2017). Long-term safety of human retinal progenitor cell transplantation in retinitis pigmentosa patients. *Stem. Cell Res. Ther.* 8:209. 10.1186/s13287-017-0661-8 28962643 PMC5622579

[B31] Lorenz B. Strohmayr E. Zahn S. Friedburg C. Kramer M. Preising M. (2012). Chromatic pupillometry dissects function of the three different light-sensitive retinal cell populations in RPE65 deficiency. *Invest. Ophthalmol. Vis. Sci.* 53 5641–5652. 10.1167/iovs.12-9974 22807296

[B32] Lu Q. Ganjawala T. H. Krstevski A. Abrams G. W. Pan Z. H. (2020). Comparison of AAV-mediated optogenetic vision restoration between retinal ganglion cell expression and ON bipolar cell targeting. *Mol. Ther. Methods Clin. Dev.* 18 15–23. 10.1016/j.omtm.2020.05.009 32548211 PMC7287188

[B33] McDougal D. H. Gamlin P. D. (2010). The influence of intrinsically-photosensitive retinal ganglion cells on the spectral sensitivity and response dynamics of the human pupillary light reflex. *Vis. Res.* 50 72–87. 10.1016/j.visres.2009.10.012 19850061 PMC2795133

[B34] Najjar R. P. Sharma S. Atalay E. Rukmini A. V. Sun C. Lock J. Z. (2018). Pupillary responses to full-field chromatic stimuli are reduced in patients with early-stage primary open-angle glaucoma. *Ophthalmol.* 125 1362–1371. 10.1016/j.ophtha.2018.02.024 29573814

[B35] Nuzbrokh Y. Ragi S. D. Tsang S. H. (2021). Gene therapy for inherited retinal diseases. *Ann. Trans. Med.* 9:1278. 10.21037/atm-20-4726 34532415 PMC8421966

[B36] Okawa H. Sampath A. P. (2007). Optimization of single-photon response transmission at the rod-to-rod bipolar synapse. *Physiology* 22 279–286. 10.1152/physiol.00007.2007 17699881

[B37] Park J. C. McAnany J. J. (2015). Effect of stimulus size and luminance on the rod-, cone-, and melanopsin-mediated pupillary light reflex. *J. Vis.* 15:13. 10.1167/15.3.13 25788707 PMC4365915

[B38] Park J. C. Moura A. L. Raza A. S. Palmer N. Gavazi E. Tsang S. H. (2010). Toward a clinical protocol for assessing rod, cone and melanopsin contributions to the human pupil response. *Invest. Ophthalmol. Vis. Sci.* 51 6624–6635. 10.1167/iovs.11-7586 PMC3175993 21743008

[B39] Park J. C. Moura A. L. Raza A. S. Rhee D. W. Kardon R. H. Hood D. C. (2011). Toward a clinical protocol for assessing rod, cone, and melanopsin contributions to the human pupil response. *Invest. Ophthalmol. Vis. Sci.* 52 6624–6635. 10.1167/iovs.11-7586 21743008 PMC3175993

[B40] Pfeiffer R. L. Marc R. E. Jones B. W. (2020). Persistent remodeling and neurodegeneration in late-stage retinal degeneration. *Prog. Retin. Eye Res.* 74:100771. 10.1016/j.preteyeres.2019.07.004 31356876 PMC6982593

[B41] Rukmini A. V. Milea D. Gooley J. J. (2019). Chromatic pupillometry methods for assessing photoreceptor health in retinal and optic nerve diseases. *Front. Neurol.* 10:76. 10.3389/fneur.2019.00076 30809186 PMC6379484

[B42] Santos A. Humayun M. S. de Juan E. Greenburg R. J. Marsh M. J. Klock I. B. (1997). Preservation of the inner retina in retinitis pigmentosa, a morphometric analysis. *Arch. Ophthalmol.* 115 511–515. 10.1001/archopht.1997.01100150513011 9109761

[B43] Schnapf J. L. Kraft T. W. Nunn B. J. Baylor D. A. (1988). Spectral sensitivity of primate photoreceptors. *Vis. Neurosci.* 1 255–261. 10.1017/s0952523800001917 3154798

[B44] Semo M. Coffey P. Gias C. Vugler A. (2016). Retrograde melanopsin signaling increases with age in retinal degenerate mice lacking rods and the majority of cones. *Invest. Ophthalmol. Vis. Sci.* 57 115–125. 10.1167/iovs.15-17609 26780315

[B45] Sharma A. Jaganathan B. G. (2021). Stem cell therapy for retinal degeneration: The evidence to date. *Biologics* 15 299–306. 10.2147/BTT.S290331 34349498 PMC8327474

[B46] Spitschan M. Jain S. Brainard D. H. Aguirre G. K. (2014). Opponent melanopsin and S-cone signals in the human pupillary light response. *Proc. Natl. Acad. Sci. U.S.A.* 111 15568–15572. 10.1073/pnas.1400942111 25313040 PMC4217411

[B47] Stingl K. T. Kuehlewein L. Weisschuh N. Biskup S. Cremers F. P. M. Khan M. I. (2019). Chromatic full-field stimulus threshold and pupillography as functional markers for late-stage, early-onset retinitis pigmentosa caused by CRB1 mutations. *Trans. Vis. Sci. Technol.* 8:45. 10.1167/tvst.8.6.45 31879567 PMC6927735

[B48] Vugler A. A. Joseph A. Jeffery G. (2008a). Survival and remodeling of melanopsin cells during retinal dystrophy. *Vis. Neurosci.* 25 125–138. 18442436 10.1017/S0952523808080309

[B49] Vugler A. A. Semo M. Joseph A. Jeffery G. (2008b). Survival and remodeling of melanopsin cells during retinal dystrophy. *Vis. Neurosci.* 25 125–138. 10.1017/S0952523808080309 18442436

[B50] Wagner J. E. Zobel L. Gerhardt M. J. O’Riordan C. R. Frederick A. Petersen-Jones S. M. (2021). In vivo potency testing of subretinal rAAV5. hCNGB1 Gene Therapy in the Cngb1 knockout mouse model of retinitis pigmentosa. *Hum. Gene Ther.* 32 1158–1170. 10.1089/hum.2021.121 34376057 PMC8819509

[B51] Yamamoto M. Matsuyama T. Maeda T. Takagi S. Motozawa N. Sakai D. (2023). Detailed evaluation of chromatic pupillometry and full-field stimulus testing to assess ultralow vision in retinitis pigmentosa. *Ophthalmol. Sci.* 3:100328. 10.1016/j.xops.2023.100328 PMC10618823 37920419

[B52] Yeh C. Y. Koehl K. L. Harman C. D. Iwabe S. Guzman J. M. Petersen-Jones S. M. (2017). Assessment of rod, cone, and intrinsically photosensitive retinal ganglion cell contributions to the canine chromatic pupillary response. *Invest. Ophthalmol. Vis. Sci.* 58 65–78. 10.1167/iovs.16-19865 28061512 PMC5231906

[B53] Yoon C. K. Yu H. G. (2018). Ganglion cell-inner plexiform layer and retinal nerve fibre layer changes within the macula in retinitis pigmentosa: A spectral domain optical coherence tomography study. *Acta Ophthalmol.* 96 e180–e188. 10.1111/aos.13577 29098796

[B54] Zhang M. Ouyang W. Wang H. Meng X. Li S. Yin Z. Q. (2021). Quantitative assessment of visual pathway function in blind retinitis pigmentosa patients. *Clin. Neurophysiol.* 132 392–403. 10.1016/j.clinph.2020.11.023 33450562

