# Supplementary material for: Chromatic pupillometry isolation and evaluation of intrinsically photosensitive retinal ganglion cell-driven pupillary light response in patients with retinitis pigmentosa
Source: Front Hum Neurosci. 2023 Jul 18;17:1212398. doi: 10.3389/fnhum.2023.1212398 (PMC10390747; doi:10.3389/fnhum.2023.1212398)
Supplement: Supplementary file 1 [file Table_1.DOCX]

Supplemental Table. Demographics details of advanced RP patients.

| Patient | Sex | Age (y) | Eye | Visual Acuity | Visual field | ERG |
| --- | --- | --- | --- | --- | --- | --- |
| RP 1 | F | 30 | R | NLP | 0° | ND |
|  |  |  | L | NLP | 0° | ND |
| RP 2 | M | 36 | L | NLP | 0° | ND |
| RP 3 | F | 47 | R | NLP | 0° | ND |
| RP 4 | M | 58 | R | NLP | 0° | ND |
|  |  |  | L | NLP | 0° | ND |
| RP 5 | M | 49 | L | NLP | 0° | ND |
| RP 6 | F | 58 | R | NLP | 0° | ND |
|  |  |  | L | NLP | 0° | ND |
| RP 7 | M | 21 | R | LP | 0° | ND |
|  |  |  | L | LP | 0° | ND |
| RP 8 | M | 43 | R | LP | 0° | ND |
|  |  |  | L | LP | 0° | ND |
| RP 9 | F | 43 | R | LP | 0° | ND |
|  |  |  | L | LP | 0° | ND |
| RP 10 | F | 39 | R | LP | 0° | ND |
|  |  |  | L | LP | 0° | ND |
| RP 11 | M | 44 | L | LP | 0° | ND |
| RP 12 | F | 28 | L | LP | 0° | ND |
| RP 13 | M | 56 | R | LP | 0° | ND |
| RP 14 | M | 36 | R | LP | 0° | ND |
| RP 15 | M | 59 | R | LP | 0° | ND |
|  |  |  | L | LP | 0° | ND |
| RP 16 | F | 60 | R | LP | 0° | ND |
|  |  |  | L | LP | 0° | ND |
| RP 17 | M | 49 | R | LP | 0° | ND |
|  |  |  | L | LP | 0° | ND |
| RP 18 | M | 33 | L | LP | 0° | ND |
| RP 19 | M | 55 | L | HM | < 10° | ND |
| RP 20 | M | 21 | R | HM | < 10° | ND |
|  |  |  | L | HM | < 10° | ND |
| RP 21 | M | 46 | R | HM | < 10° | ND |
| RP 22 | F |  | R | HM | < 10° | ND |
| RP 22 | F | 51 | L | HM | < 10° | ND |
| RP 23 | F | 28 | R | HM | < 10° | ND |
| RP 24 | M | 39 | R | HM | < 10° | ND |
| RP 25 | F | 47 | L | HM | < 10° | ND |
| RP 26 | F | 48 | R | HM | < 10° | ND |
|  |  |  | L | HM | < 10° | ND |
| RP 27 | M | 54 | L | HM | < 10° | ND |
| RP 28 | F | 48 | L | HM | < 10° | ND |
| RP 29 | F | 40 | L | HM | < 10° | ND |
| RP 30 | F | 33 | R | HM | < 10° | ND |
|  |  |  | L | HM | < 10° | ND |
| RP 31 | F | 56 | R | HM | < 10° | ND |
|  |  |  | L | HM | < 10° | ND |
| RP 32 | F | 49 | L | HM | < 10° | ND |
|  |  |  | R | HM | < 10° | ND |
| RP 33 | M | 27 | R | HM | < 10° | ND |
| RP 34 | M | 58 | R | HM | < 10° | ND |
|  |  |  | L | HM | < 10° | ND |
| RP 35 | F | 50 | L | HM | < 10° | ND |
|  |  |  | R | HM | < 10° | ND |
| RP 36 | M | 38 | R | HM | < 10° | ND |
|  |  |  | L | HM | < 10° | ND |
| RP 37 | F | 59 | L | HM | < 10° | ND |
|  |  |  | R | HM | < 10° | ND |
| RP 38 | M | 56 | R | CF | < 10° | ND |
|  |  |  | L | CF | < 10° | ND |
| RP 39 | F | 60 | L | CF | < 10° | ND |
| RP 40 | M | 32 | R | CF | < 10° | ND |
|  |  |  | L | CF | < 10° | ND |
| RP 41 | M | 40 | L | 0.01 | < 10° | ND |
| RP 42 | M | 36 | L | 0.01 | < 10° | ND |
| RP 43 | F | 46 | R | 0.02 | < 10° | ND |
|  |  |  | L | 0.02 | < 10° | ND |
| RP 44 | M | 43 | R | 0.03 | < 10° | ND |
| RP 45 | M | 37 | R | 0.04 | < 10° | ND |
| RP 46 | M | 27 | L | 0.04 | < 10° | ND |
|  |  |  | R | 0.04 | < 10° | ND |
| RP 47 | M | 36 | L | 0.05 | < 10° | ND |
| RP 48 | M | 39 | L | 0.05 | < 10° | ND |
| RP 49 | M | 44 | R | 0.05 | < 10° | ND |
| RP 50 | M | 29 | R | 0.1 | < 10° | ND |
|  |  |  | L | 0.1 | < 10° | ND |
| RP 51 | M | 44 | R | 0.1 | < 10° | ND |
| RP 52 | M | 46 | L | 0.1 | < 10° | ND |
|  |  |  | R | 0.2 | < 10° | ND |
| RP 53 | F | 45 | L | 0.1 | < 10° | ND |
|  |  |  | R | 0.5 | < 10° | ND |
| RP 54 | M | 35 | L | 0.1 | < 10° | ND |
| RP 55 | M | 33 | R | 0.15 | < 10° | ND |
|  |  |  | L | 0.15 | < 10° | ND |
| RP 56 | F | 30 | R | 0.15 | < 10° | ND |
|  |  |  | L | 0.2 | < 10° | ND |
| RP 57 | F | 50 | R | 0.15 | < 10° | ND |
| RP 58 | M | 35 | R | 0.15 | < 10° | ND |
| RP 59 | M | 34 | R | 0.2 | < 10° | ND |
|  |  |  | L | 0.3 | < 10° | ND |
| RP 60 | M | 46 | L | 0.2 | < 10° | ND |
| RP 61 | F | 40 | R | 0.2 | < 10° | ND |
| RP 62 | F | 47 | L | 0.2 | < 10° | ND |
| RP 63 | F | 44 | R | 0.2 | < 10° | ND |
| RP 64 | F | 27 | R | 0.2 | < 10° | ND |
| RP 65 | M | 44 | R | 0.2 | < 10° | ND |
|  |  |  | L | 0.4 | < 10° | ND |
| RP 66 | F | 55 | R | 0.3 | < 10° | ND |
|  |  |  | L | 0.5 | < 10° | ND |
| RP 67 | M | 49 | L | 0.3 | < 10° | ND |
|  |  |  | R | 0.3 | < 10° | ND |

RP: retinitis pigmentosa, NLP: no light perception, LP: light perception, HM: hand movement, CF: counting finger, ERG: electroretinogram, ND: nondetectable
